# Immunohistochemical analysis of cancer stem cell markers in pancreatic adenocarcinoma patients after neoadjuvant chemoradiotherapy

**DOI:** 10.1186/1471-2407-14-687

**Published:** 2014-09-21

**Authors:** Tatsuzo Mizukami, Hirofumi Kamachi, Tomoko Mitsuhashi, Yosuke Tsuruga, Yutaka Hatanaka, Toshiya Kamiyama, Yoshihiro Matsuno, Akinobu Taketomi

**Affiliations:** Department of Gastoroenterological Surgery I, Graduate School of Medicine, Hokkaido University, North 15, West 7, Kita-ku, Sapporo, 060-8638 Japan; Department of Surgical Pathology, Hokkaido University Hospital, North 14, West 5, Kita-ku, Hokkaido, Sapporo, 060-8648 Japan

**Keywords:** Cancer stem cells, EpCAM, CD24, CD44, CD133, CXCR4, ALDH1, Neoadjuvant chemoradiotherapy, Pancreatic cancer

## Abstract

**Background:**

Cancer stem cells (CSCs) have been reported to play an important role in chemoradiation resistance. Although the association of CSC markers with clinicopathological outcomes after neoadjuvant chemoradiotherapy (NACRT) has been reported in various types of cancers, there have been no such reports for pancreatic cancer. Here we examined the sequential changes in CSC marker expressions after NACRT in patients with pancreatic adenocarcinoma (PA) and the impact of these changes on the prognosis.

**Methods:**

We used immunohistochemistry to evaluate the expressions of the CSC markers epithelial cell adhesion molecule (EpCAM), CD24, CD44, CD133, CXCR4 and Aldehyde dehydrogenase 1 (ALDH1) in resected specimens obtained from 28 PA patients, and we compared these expressions with the patients’ clinicopathological parameters and survival data.

**Results:**

The expression frequencies of CD44 and ALDH1 were significantly higher in the NACRT group (n = 17) compared to the non-NACRT group (n = 11), but the CD133 expression was significantly lower in the NACRT group. In the NACRT group, the expression of CD133 was inversely correlated with that of ALDH1, and CD133+/ALDH1- expression was associated with an unfavorable patient outcome.

**Conclusion:**

This is the first report showing that NACRT may influence the expression frequencies of CD44, CD133 and ALDH1 in PA patients. Moreover, CD133 and ALDH1 expressions may be useful predictors of prognosis in PA patients who have received NACRT.

**Electronic supplementary material:**

The online version of this article (doi:10.1186/1471-2407-14-687) contains supplementary material, which is available to authorized users.

## Background

Pancreatic cancer is the fourth leading cause of cancer death in the United States, and its 5-year survival rate is only 6% [1]. Surgical resection remains the only potentially curative therapeutic option. However, pancreatic cancer proceeds asymptomatically in many cases, and surgical resection is feasible in only 10% to 20% of patients at the time of initial diagnosis [2]. Even after complete resection, the long-term survival rate remains very poor [3, 4].

New therapeutic strategies are thus needed to improve the prognosis of pancreatic cancer patients. During the past decade, neoadjuvant chemoradiotherapy (NACRT) for locally advanced pancreatic adenocarcinoma has received attention [5]. NACRT has several positive aspects such as an increased resectability rate with clear margins and decreased rates of metastatic lymph nodes and local relapse, and NACRT resulted in a significant improvement of the 5-year survival rate in curative cases [6, 7]. However, many patients with pancreatic cancer do not respond to NACRT, and little is known about the potential biological markers that may be associated with response to NACRT.

Evidence has accumulated indicating that many solid tumours are driven and managed by rare subpopulations of cancer stem cells (CSCs). In pancreatic cancer, several markers have been used to identify CSCs, such as epithelial cell adhesion molecule (EpCAM, also known as epithelial-specific antigen, or ESA) [8], CD24 [9], CD44 [10, 11], CD133 [12, 13], CXCR4 [14], aldehyde dehydrogenase 1 (ALDH1) [15, 16] and combinations of these markers [17–19]. And it has been reported that the expression of CSCs related to patients prognosis [20]. The biological roles of each CSCs marker are widely different. EpCAM is considered an adhesion molecule. CD24 and CD44 also function as adhesion molecules. CD133 is a cell surface glycoprotein. CXCR4 functions as a chemokine receptor. ALDH1 is an intracellular enzyme involved in retinoic acid.

CSCs seem to be primarily responsible for the frequently observed failure of therapies as well as for relapse after anticancer treatment [21]. In fact, there are several reports of the resistance of CSCs to chemoradiation therapy in head-neck [22], esophageal [23, 24], lung [25] and colon [26] cancer, but there has been no report on pancreatic CSCs related to chemoradiation resistance, to our knowledge.

In the present study therefore, we investigated the properties of pancreatic CSCs to compare the expressions of CSC markers in the tumours of PA patients according to whether they received NACRT, and to analyze the associations between the expressions of the CSC markers and the clinicopathological characteristics of the NACRT group to determine the clinical implications of the CSC marker expressions.

## Methods

### Patient demographics

Between May 2003 and September 2013, 28 PA patients (14 males, 14 females) underwent surgery at the Department of General Surgery I, Hokkaido University Graduate School of Medicine (Sapporo, Japan). Among them, 17 patients received preoperative chemoradiotherapy with gemcitabine (GEM) followed by 50.4 Grays (Gy) of radiation therapy (NACRT group). All patients in the NACRT group received a cumulative irradiation dose of 50.4 Gy in 28 fractions of 1.8 Gy, using 3-dimensional radiation therapy. The primary tumour plus regional lymph nodes were targeted. Systemic GEM 150 mg/m^2^ was administered weekly. Within 4–6 weeks after the completion of NACRT, the patients were reassessed by CT, MRI and PET-CT and surgery was performed. During the same period, 11 patients did not receive preoperative chemoradiotherapy but underwent surgery (the non-NACRT group).

Recurrence was diagnosed on the basis of clinical examinations and imaging studies. Time to death, final follow-up examination, and the diagnosis of recurrence was measured from the date of surgery. Surviving patients were followed up until March 2014.

Written informed consent was obtained from all 28 patients prior to their enrollment in the study, and this study design and protocol were approved by the institutional review board of Hokkaido University Hospital Sapporo, Japan (Clinical Research approval number 013–0074).

### Pathological specimens

Formalin-fixed and paraffin-embedded specimens were retrieved from the surgical pathology files of the Pathology Department of Hokkaido University Hospital. Sections were cut and stained with hematoxylin-eosin (H&E) for routine histopathologic examination. Pancreatic ductal adenocarcinoma was diagnosed in all specimens. A representative tissue block was selected from each case to perform immunohistochemical studies.

### Immunohistochemistry

The resected tissues were fixed in 10% formalin and embedded in paraffin blocks, and the most representative block was chosen for each case. Each block was cut into serial 4-μm-thick sections for staining with H&E and immunohistochemistry for EpCAM, CD24, CD44, CD133, CXCR4 and ALDH1. Immunohistochemistry was performed using the EnVision + System-HRP (Dako Japan, Tokyo), and the protocol was optimized for each antigen (Table 1).

**Table 1:** **Primary antibodies used in the immunohistochemistry**

Antigen (clone)	Location	Antibody species	Manufacturer (product)	Antigen-retrieval solution	Dilution	Internal positive control [reference No]
EpCAM (ESA)	M	Mouse monoclonal	Dako (M3525)	PH6 ciltrate buffer	1:200	Epithelium of pancreatic ducts, acinar cells and islets of Langerhans cells [29]
CD24	M	Mouse monoclonal	Neomarkers (MS-1279)	PH6 ciltrate buffer	1:50	Acinar cells [27]
CD44	M	Mouse monoclonal	Abcam(ab51037)	PH6 ciltrate buffer	1:50	Acinar cells [28]
CD133	M	Rabbit polyclonal	Abnova(12663)	PH9 Tris EDTA buffer	1:50	Acinar cells [19]
CXCR4	M	Mouse monoclonal	Zymed(35-8800)	PH6 ciltrate buffer	1:50	Acinar cells [17]
ALDH1	C	Mouse monoclonal	Abcam(ab52492)	PH6 ciltrate buffer	1:100	Acinar cells and islets of Langerhans cells [19]

M: membrane C: cytoplasm.

Briefly, the sections were mounted on charged glass slides, deparaffinized, and rehydrated through a graded ethanol series. Antigens were retrieved in Dako EnVision FLEX Target Retrieval Solution using Dako PT Link for 20 min at 97°C according to the manufacturer’s instructions (Dako Japan). After the blocking of endogenous peroxidase activity with 0.03% hydrogen peroxide, the sections were incubated with the primary antibodies at room temperature for 30 min and then reacted with a dextran polymer reagent combined with secondary antibodies and peroxidase for 30 min at room temperature. Specific antigen-antibody reactions were visualized with diaminobenzidine chromogen applied for 10 min. Slides were counterstained with hematoxylin, dehydrated and mounted.

Non-neoplastic pancreatic tissues on the same slides as those summarized in Table 1 were defined as internal positive controls for each antibody [17, 19, 27–29]. Negative control tissue sections were prepared by omitting the primary antibody.

### Immunohistochemical evaluation

All assessments were made on the tumour region of the specimen (×200). Each slide was evaluated independently by two independent observers (authors TM and TM), who did not know the clinical outcomes, and discrepancies between the observers were resolved using a conference microscope. To take into account intratumoral heterogeneity of antigen expression, we selected two to six visual fields from different areas of invasive ductal carcinoma excluding that of intraepithelial neoplasia in each slide. In detail, guided by the microscope, the areas were selected randomly per section using a × 4 objective and a × 10 ocular lens on each H-E staining slide and marked it by circling each area. And then, we superimposed the slide which was stained with CSCs markers on the HE staining slide, and have marked it by tracing the mark for evaluation of the immunoreacting score (IRS). The immunoreaction for each antibody was evaluated in each case based on both the proportion of positive-stained tumour cells and the staining intensity of the tumour cells . The expression site of each antibody (membrane or cytoplasm) is given in Table 1. The expression of each antibody was evaluated for each tissue sample by calculating the total IRS as the product of the proportion and intensity scores according to previous reported criteria [22]. Briefly, the proportion score reflected the estimated fraction of positive-stained tumour cells (0, none; 1, 1%–10%; 2, 11%–50%; 3, 51%–80%; 4, 81%–100%). The intensity score represented the estimated staining intensity (0, no staining; 1, weak; 2, moderate; 3, strong). The total IRS ranged from 0 to 12, and the scores were averaged. A positive expression of each antibody was defined as an averaged score > median.

### Statistical analysis

We used a t-test or Fisher’s exact test to evaluate the differences in clinicopathological and immunohistological features between the NACRT and non-NACRT group. We tested the associations between clinicopathological and immunohistologically detected stem cell marker expressions by Fisher’s exact test. Survival curves of patients were drawn by the Kaplan- Meier method. Differences in survival curves were analyzed by the log-rank test. Differences at P < 0.05 were considered significant. All statistical analyses were performed using JMP Pro 10 (SAS Institute Japan).

## Results

### Patient characteristics

The patient demographics are shown in Table 2. T-factor, N-factor, Histological classification and R-factor were assigned according to the TNM classification of the Union Internationale Contre le Cancer (UICC 7th edition). There were no significant differences between the NACRT and non-NACRT groups in age, gender, operative procedures, portal vein resection, clinical T, N factor, pathological T, N factor, histological classification, or residual tumour.

**Table 2:** **Patient demographics and clinicpathological characteristics**

	NACRT (n = 17)	Non-NACRT (n = 11)	P-value
Age(mean ± SD)	59.9 ± 7.9	63.6 ± 10.4	0.287^*1^
Gender(male/female)	8/9	6/5	1.000^*2^
Operative procedures PD/DP/TP	13/3/1	10/1/0	1.000^*2^
Portal vein resection	70.6% (12/17)	63.6% (7/11)	1.000^*2^
cT(1/2/3/4)	0/0/17/0	0/0/11/0	1.000^*2^
cN(0/1)	11/6	6/5	0.701^*2^
pT(0/1/2/3/4)	0/1/16/0	0/0/11/0	1.000^*2^
pN(0/1)	13/4	5/6	0.125^*2^
Histological classification G1-2/G3/ungradeable	11/5/1	8/3/0	1.000^*2^
Residual tumor R0/R1-2	17/0	9/2	0.146^*2^
Tumor destruction (Evan’s criteria)I/IIa/IIb/III/IV	1/7/7/2/0		

SD: standard deviation; PD: pancreatoducdenectomy; DP: distal pancreatectomy; TP total pancreatectomy. *1: Unpaired t-test. *2: Fisher’s exact test.

In the evaluation of tumour destruction, over 50% of the cancer cells had degenerated in nine patients.

### Patterns of expression

**Figure 1:**
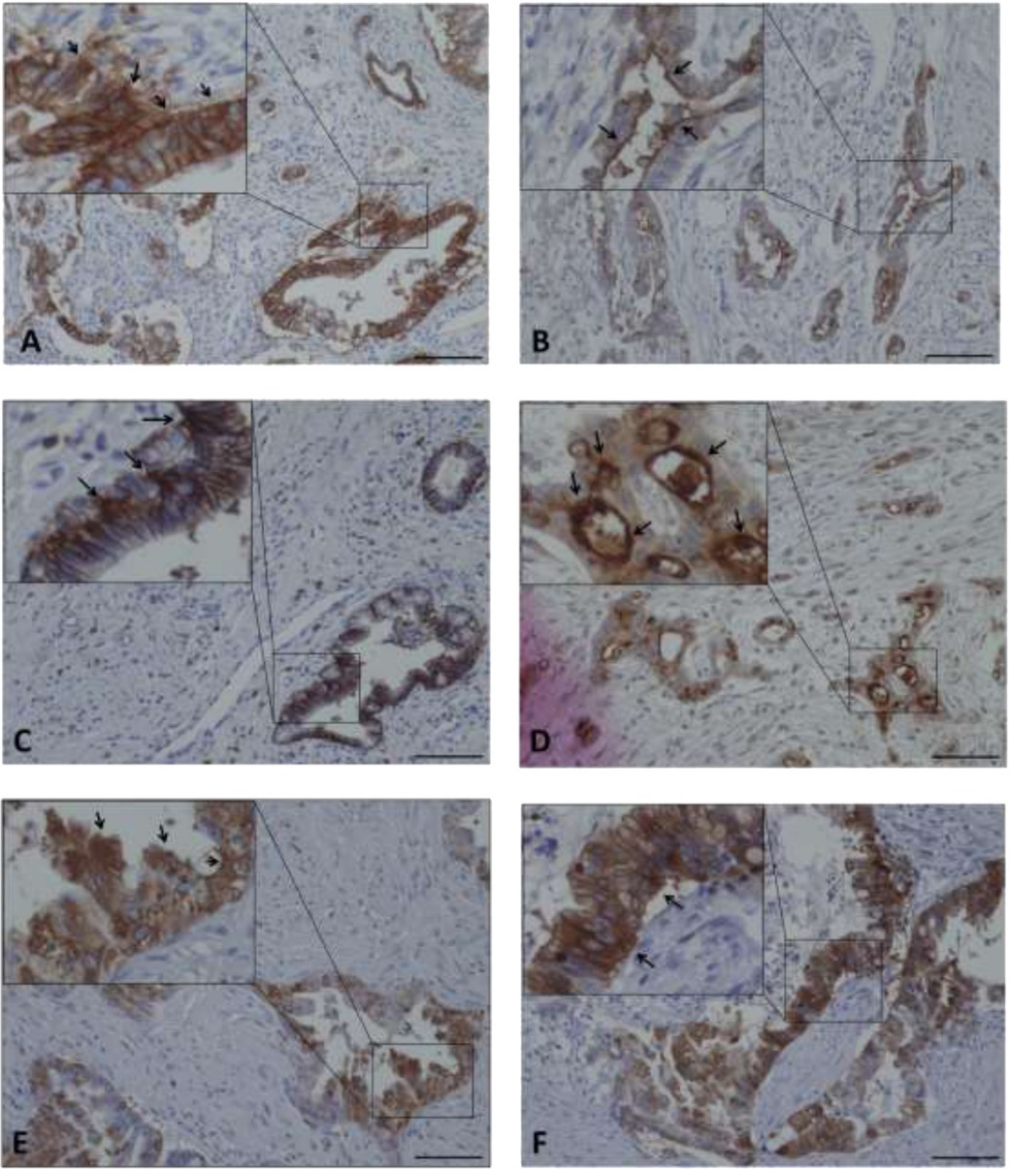
**Immunohistochemical staining of each CSC marker in pancreatic adenocarcinoma.** The arrows indicate strong staining intensity of EpCAM **(A)**, CD24 **(B)**, CD44 **(C)**, CD133 **(D)**, CXCR4 **(E)** and ALDH1 **(F)**. Scale bar, 100 μm.

The expressions of EpCAM, CD24, CD44, CD133, and CXCR4 antigens were membranous in carcinoma cells (Figure 1A–E).

The IRS of cancer cells with membranous EpCAM expression ranged from 0 to 12 (median 7.3). Using the cutoff point, of the 28 cases, 15 (53.6%) were considered positive. The IRS of the cancer cells with membranous CD44 expression ranged from 0 to 12 (median 3.4), with 16/28 (57.1%) cases considered positive. The IRS of the cancer cells with membrane CD24 expression ranged from 0 to 12 (median 2.9), with 10/28 (35.7%) cases being positive. The IRS of the cancer cells with membrane CD133 expression ranged from 1 to 12 with a median value of 5.7, and 15/28 (53.6%) cases were considered positive for CD133. The IRS of the cancer cells with membranous CXCR4 expression ranged from 1 to 12 (median 6.1), with 11/28 (39.3%) cases considered positive.

The expression of ALDH1 was cytoplasmic in carcinoma cells (Figure 1F). The IRS of the cancer cells with cytoplasmic ALDH1 expression ranged from 1 to 12 with a median value of 5.6. Of the 28 cases, 13 (46.4%) were considered positive.

### Response analysis

As shown in Table 3, a positive CD44 expression was found in 14 of the 17 cases (82.4%) in the NACRT group and in 2 of the 11 cases (18.2%) in the non-NACRT group; the difference between the two groups was significant (P = 0.00148).

**Table 3:** **Frequency of CSCs markers positive cases**

	NACRT(n = 17)	Non-NACRT(n = 11)	P-value
EpCAM(+)	58.8%	45.5%	0.700
CD24(+)	35.3%	36.4%	1.000
CD44(+)	82.4%	18.2%	**0.00148**
CD133(+)	29.4%	81.8%	**0.0183**
CXCR4(+)	47.1%	27.2%	0.435
ALDH1(+)	64.7%	18.2%	**0.0237**

Fisher’s exact test.

The bold value indicates a statistically significant result.

Positive CD133 expression was found in 5 of the 17 cases (29.4%) in the NACRT group and in 9 of the 11 cases (81.8%) in the non-NACRT group; the difference between the two groups was significant (P = 0.0183).

Positive ALDH1 expression was found in 11 of the 17 cases (64.7%) in the NACRT group and in 2 of the 11 cases (18.2%) in the non-NACRT group; the difference between the two groups was significant (P = 0.0237).

No significant differences were found in the frequency of expression of EpCAM, CD24 or CXCR4 between the NACRT group and the non-NACRT group.

### Correlation among CSC markers

As shown in Table 4, CD133 expression was inversely related to ALDH1 expression in the NACRT group (P = 0.0276), but no significant associations were observed between the other CSCs markers.

**Table 4:** **Correlations between CSC marker expressions in the NACRT group**

	ESA			CD24			CD44			CD133			CXCR4		
	(+)	(-)	P	(+)	(-)	P	(+)	(-)	P	(+)	(-)	P	(+)	(-)	P
CD24															
(+)	4	2	1.000												
(-)	6	5													
CD44															
(+)	9	5	0.537	4	10	0.515									
(-)	1	2		2	1										
CD133															
(+)	3	2	1.000	3	2.280	5	0	0.515							
(-)	7	5		3	9	9	3								
CXCR4															
(+)	4	4	0.637	4	4	0.335	6	2	0.576	2	6	1.000			
(-)	6	3		2	7		8	1		3	6				
ALDH1															
(+)	7	4	0.644	3	8	0.600	9	2	1.000	1	10	**0.0276**	5	6	1.000
(-)	3	3		3	3		5	1		4	2		3	3	

Fisher’s exact test.

The bold value indicates a statistically significant result.

### Association with histopathological variables

Table 5 shows the associations of CSC markers with clinicopathologic features in the NACRT group. The positive expression of CXCR4 was significantly correlated with a higher liver metastasis rate (P = 0.0152).

**Table 5:** **Association of CSC markers with clincopathologic features in the NACRT group**

		EpCAM (+)		CD24 (+)		CD44 (+)		CD133 (+)		CXCR4 (+)		ALDH1 (+)	
Parameter	Total	(n = 10)	p	(n = 6)	p	(n = 14)	p	(n = 5)	p	(n = 8)	p	(n = 11)	p
Histological classification													
Grade 1/2	11	8	0.186	4	0.547	9	1.000	4	0.0801	3	0.0633	8	0.547
Grade 3	5	2		1		4		0		4		3	
_ungradeable_	1	0		1		1		1		1		0	
ypT													
ypT2	1	1	1.000	0	1.000	1	1.000	0	1.000	0	1.000	1	1.000
ypT3	16	9		6	13			5		8		10	
ypN													
ypN1	4	3	0.603	2	0.584	3	1.000	2	0.538	2	1.000	3	1.000
ypN0	13	7		4		11		3		6		8	
Tumour down stage													
Present	5	4	0.338	2	1.000	5	0.515	1	1.000	4	0.131	3	1.000
Absent	12	6		4		9		4		4		8	
Lymphatic invasion													
Present	1	0	1.000	0	1.000	1	1.000	0	1.000	0	1.000	1	1.000
Absent	16	10		6		13		5		8		10	
Blood vessel invasion													
Present	11	7	0.644	3	0.600	9	1.000	3	1.000	4	0.335	7	1.000
Absent	6	3		3		5		2		4		4	
Perineural invasion													
Present	12	6	0.338	3	0.280	10	1.000	3	0.600	6	1.000	8	1.000
Absent	5	4		3		4		2		2		3	
Recurrence													
Present	11	6	1.000	5	0.333	10	0.515	4	0.600	7	0.131	7	1.000
Absent	6	4		1		4		1		1		4	
Liver metastasis													
Present	7	3	0.350	3	0.644	6	1.000	2	1.000	6	**0.0152**	6	0.304
Absent	10	7		3		8		3		2		5	
Tumor destruction (Evans’s criteria)													
I/lla	8	5	1.000	1	0.131	7	1.000	1	0.294	3	0.637	5	1.000
llb/lll	9	5		5		7		4		5		6	

Fisher’s exact test.

The bold value indicates a statistically significant result.

### Positive CD133 and negative ALDH1 expression had a markedly poorer OS

**Figure 2:**
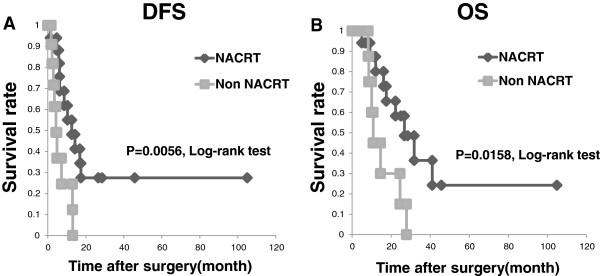
**Prognostic analysis of NACRT. A**: Disease-free survival (DFS) of the patients stratified by the treatment method. The DFS of the patients with NACRT was significantly better than that of the non-NARCT patients (median DFS 12.6 mos for the NACRT group vs. 4.3 mos for the non-NACRT group; P = 0.0056). **B**: Overall survival (OS) for patients stratified by the treatment method. The OS of the NACRT group was significantly better than that of the non-NACRT group (median OS 26.8 mos for the NACRT group vs. 10.8 mos for the non-NACRT group; P = 0.0158).

Figure 2 shows that the patients who underwent NACRT had significantly better disease-free survival (DFS) and overall survival (OS) rates compared to the patients who did not undergo NACRT (P = 0.0056 and P = 0.0158, respectively).

**Figure 3:**
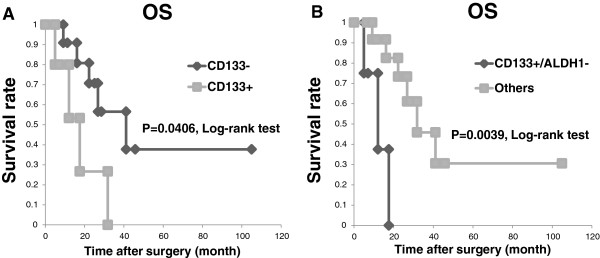
**Positive CD133 expression correlates with poor prognosis in the NACRT group. A**: The OS of the NACRT patients stratified by their CD133 expression status. The OS of the patients with positive CD133 expression was significantly worse than that of the patients with negative CD133 expression (median OS 17.6 mos for those showing positive CD133 expression vs. 41.1 mos for those showing negative CD133 expression; P = 0.0406). **B**: Overall survival for the NACRT patients stratified by their CD133 and ALDH1 expression status. The OS of the patients with CD133+/ALDH1- was significantly worse than that of the patients with the other markers’ expressions (median OS 12.1 mos for those showing CD133+/ALDH1- vs. 31.8 mos for those showing the other markers’ expressions; P = 0.0039).

In the NACRT group, the patients with positive CD133 expression had a significantly poorer OS rate (P = 0.0406) compared to those with negative CD133 expression (Figure 3A).

However, the patients with positive expression of CD44 and ALDH1 had no significant differences in prognosis compared to the patients with negative expression of CD44 and ALDH1. In addition, the patients with positive CD133 and negative ALDH1 expression had a markedly poorer OS rate (P = 0.0039) compared to the patients with expressions of other markers (Figure 3B).

## Discussion

In this study, we focused on EpCAM, CD24, CD44, CD133, CXCR4 and ALDH1 as representative pancreatic CSC markers and examined the effect of NACRT on pancreatic CSCs. Our major findings are as follows: (1) CD44- and ALDH1-positive cells may have chemoradiation resistance, but CD133-positive cells may have chemoradiation susceptibility in pancreatic cancer; (2) CD133 and ALDH1 expressions may be useful predictors of prognosis in pancreatic adenocarcinoma patients who have received NACRT. As the evaluation method of the effect of NACRT, we compared the expression of several CSC markers immunohistochemically detected in human pancreatic cancer specimens from patients who received and did not receive NACRT. Although the comparison of tissue samples obtained from the same individual before and after NACRT is desirable, the evaluation has been difficult in terms of the quantity and quality of biopsy material before NACRT. Thus, we think that comparisons between patients in similar cohorts who received and did not receive NACRT are adequate to determine whether the survival of CSC marker-positive cells is a phenomenon that occurs in human cancer tissue.

Regarding the chemoradiation resistance to pancreatic CSCs, we have demonstrated that the frequencies of CD44- and ALDH1-positive cases are increased in the NACRT group. This result indicates that CD44- and ALDH1-positive cells may have chemoradiation resistance in pancreatic cancer. CD44 is involved in cell-to-cell and cell-to-matrix interactions and has been used as a CSC marker in cancers of the head and neck [22], breast [30] and prostate [31]. Similar to our results, Tajima et al. [10] showed the frequencies of CD44- positive cases were increased after gemcitabine-based neoadjuvant chemotherapy and concluded CD44- positive cells were chemoresistant in pancreatic cancer.

ALDH1 is an intracellular enzyme involved in retinoic acid, and it has been characterized as a CSC marker in different types of cancer of the head and neck [22], breast [30], lung [32], and colon [33]. In pancreas cancer, ALDH1 was associated with high turmorigenic cancer cells [34], and protects pancreatic cancer cells from chmothrapy-induced cell death [35]. Two immunohistochemical studies examined the prognostic significance of ALDH1 in pancreatic cancer, but their results conflict, perhaps because the evaluation methods differed (using tissue microarrays [15] vs. whole-mount tissue slides [16]). Moreover, there were also no immunohistochemical studies about the chemoradiation resistance. Our finding is a first report indicating that ALDH1-positive cells might be resistant to chemoradiation therapy.

On the other hand, our results have also showed that CD133-positive cells may have chemoradiation susceptibility. CD133 is a cell surface glycoprotein that has been widely used as a marker for CSCs in various types of solid tumours and it has been believed that the CD133-positive cells had chmoradiation resistance [36].

One of the reasons why our data are different from published literature may be related to the antibodies we used and the number of cases, as well as to the influence of NACRT.

Also, this conflicting result can be explained under the assumption that not all CD133-positive cells are characterized as the same cell population, and not all these cells are resistant to chemoradiation. It may be that clonogenicity varies among cancer cells bearing distinct cancer stem cell markers, and so does their sensitivity to altered fractionation. In fact, it has been reported regarding the susceptibility of CD133-positive cells for chemoradiation in gastric [37] and colon cancer [38]. Additional study in larger cohorts and basic research are required to clarify this result.

Regarding the prognosis in the NACRT group, there are no significant differences in DFS (Additional file 1: Figure S1) and OS (Additional file 2: Figure S2) in almost all CSCs marker expect CD133. Despite CD133-positive cells apparently may have chemoradiation susceptibility, this data is consistent with the results that the expression CD133-positive cells in pancreatic cancer without NACRT related to poor clinical outcome [12, 13]. Thus, CD133 expression has a possibility to influence the prognosis on pancreatic cancer regardless of the presence or absence of NACRT. Furthermore, our results suggest that NACRT might reduce the frequency of CD133 expression and subsequently result in patient’s favorable prognosis in pancreatic cancer.

With respect to the CSCs markers expression, there were almost all no associations among the co-expression of different CSCs markers used in our study. Interestingly, although its significance is unknown, CD133 expression was inversely related to ALDH1 expression after NACRT, and the patients with positive CD133 and negative ALDH1 expression had a markedly poorer OS rate compared to the other patients. A similar result was reported for head and neck cancer treated with chemoradiation, in which positive CD44 and negative ALDH1 expression was linked with significantly poor prognosis [22]. ALDH1 is an enzyme that is required for the conversion of retinol (vitamin A) to retinoic acids and retinoic acid is related to the differentiation of cells, so inhibition of ALDH1 delayed the differentiation of human hematopoietic stem cells [39].

We speculate the expression of ALDH1 is also related to the differentiation of cancer stem cells.

As a result, combination with several stem cell markers may become a more powerful prognosis prediction marker.

## Conclusions

We found that CD44- and ALDH1-positive expressions were more common in the NACRT group than in the non-NACRT group, whereas CD133-positive expression was found to be common in the non-NACRT group. In addition, CD133+ expression and CD133+/ALDH1- expression were associated with a poor outcome in the NACRT group. CD133 and ALDH expressions are useful predictors of prognosis in PA patients who have received NACRT.

However, our results were obtained in a small cohort (n = 28) of PA patients, and additional studies in larger cohorts are required to clarify the predictive significance, if any, of the expressions of CSCs markers in pancreatic cancer.

## Electronic supplementary material

Additional file 1: Figure S1: Significance of the CSCs markers in Disease-free survival (DFS) in the NACRT group. The DFS of the NACRT patients stratified by their CSCs marker expression status. There are no significant differences in DFS in all CSCs marker. (PPTX 1 MB)

Additional file 2: Figure S2: Significance of the CSCs markers in Overall survival (OS) in the NACRT group. The OS of the NACRT patients stratified by their CSCs marker expression status. There are no significant differences in OS in almost allCSCs marker expect CD133. (PPTX 988 KB)

## Abbreviations

CSCs: Cancer stem cells
NACRT: Neoadjuvant chemoradiotherapy
PA: Pancreatic adenocarcinoma
EPCAM: Epithelial cell adhesion molecule
ALDH1: Aldehyde dehydrogenase 1
GEM: Gemcitabine
Gy: Grays
IRS: Immunoreacting score
DFS: Disease-free survival
OS: Overall survival.
