# Supplementary material for: Immunohistochemical analysis of cancer stem cell markers in pancreatic adenocarcinoma patients after neoadjuvant chemoradiotherapy
Source: BMC Cancer. 2014 Sep 21;14:687. doi: 10.1186/1471-2407-14-687 (PMC4190289; doi:10.1186/1471-2407-14-687)
Supplement: Supplementary file 1 — Additional file 1: Figure S1: Significance of the CSCs markers in Disease-free survival (DFS) in the NACRT group. The DFS of the NACRT patients stratified by their CSCs marker expression status. There are no significant differences in DFS in all CSCs marker. (PPTX 1 MB) [file 12885_2014_4892_MOESM1_ESM.pptx]

## Slide 1
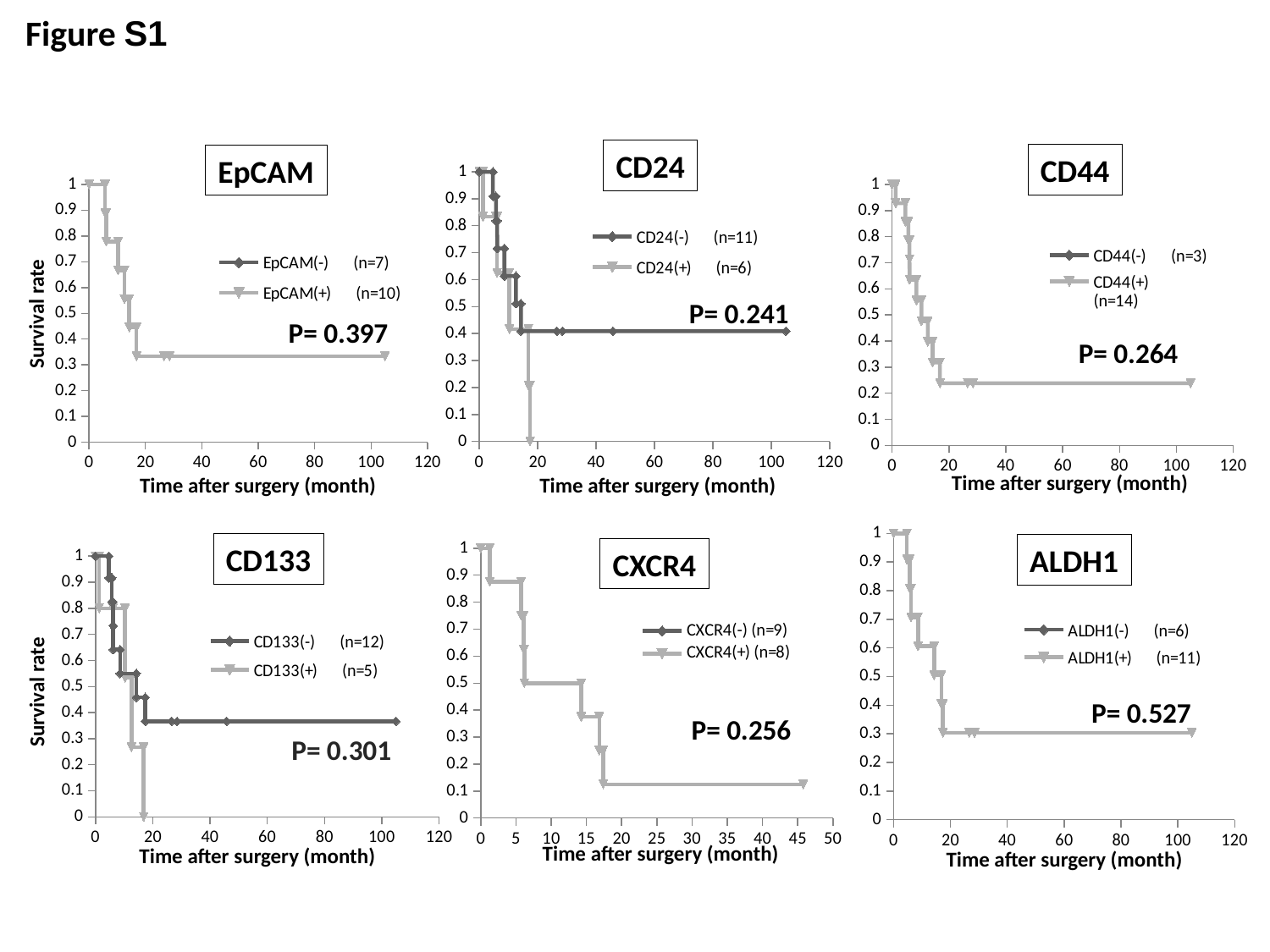

Figure S1
CD24
CD44
EpCAM
### Chart
| Category | | |
|---|---|---|
### Chart
| Category | | |
|---|---|---|
### Chart
| Category | | |
|---|---|---|P= 0.241
Survival rate
P= 0.397
P= 0.264
Time after surgery (month)
Time after surgery (month)
Time after surgery (month)
### Chart
| Category | | |
|---|---|---|CD133
### Chart
| Category | | |
|---|---|---|ALDH1
CXCR4
### Chart
| Category | | |
|---|---|---|Survival rate
P= 0.527
P= 0.256
P= 0.301
Time after surgery (month)
Time after surgery (month)
Time after surgery (month)
