# Supplementary material for: Immunohistochemical analysis of cancer stem cell markers in pancreatic adenocarcinoma patients after neoadjuvant chemoradiotherapy
Source: BMC Cancer. 2014 Sep 21;14:687. doi: 10.1186/1471-2407-14-687 (PMC4190289; doi:10.1186/1471-2407-14-687)
Supplement: Supplementary file 2 — Additional file 2: Figure S2: Significance of the CSCs markers in Overall survival (OS) in the NACRT group. The OS of the NACRT patients stratified by their CSCs marker expression status. There are no significant differences in OS in almost allCSCs marker expect CD133. (PPTX 988 KB) [file 12885_2014_4892_MOESM2_ESM.pptx]

## Slide 1
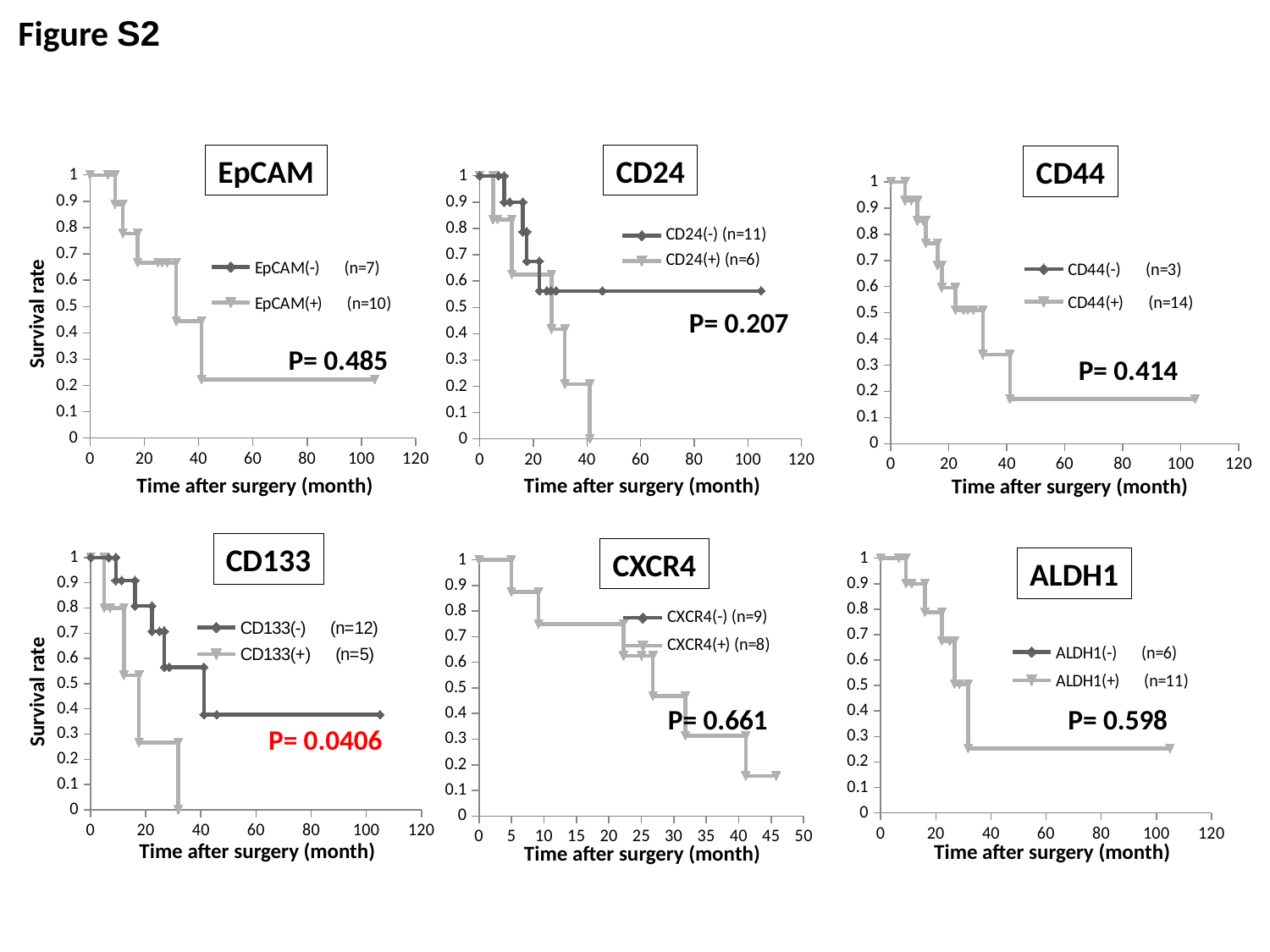

Figure S2
EpCAM
CD24
CD44
### Chart
| Category | | |
|---|---|---|
### Chart
| Category | | |
|---|---|---|
### Chart
| Category | | |
|---|---|---|Survival rate
P= 0.207
P= 0.485
P= 0.414
Time after surgery (month)
Time after surgery (month)
Time after surgery (month)
CD133
CXCR4
### Chart
| Category | | |
|---|---|---|
### Chart
| Category | | |
|---|---|---|
### Chart
| Category | | |
|---|---|---|ALDH1
Survival rate
P= 0.661
P= 0.598
P= 0.0406
Time after surgery (month)
Time after surgery (month)
Time after surgery (month)
